# Calcaneal osteomyelitis presenting with acute tarsal tunnel syndrome: a case report

**DOI:** 10.1186/1752-1947-4-66

**Published:** 2010-02-23

**Authors:** Davinder PS Baghla, Sajid Shariff, Raman Dega

**Affiliations:** 1 Hercies Road, Uxbridge, Middlesex, UB10 9LU, UK; 2 Wexham Park Hospital, Wexham Street, Slough, SL2 4HL, UK

## Abstract

**Introduction:**

Cases of acute tarsal tunnel syndrome are rare. To the best of our knowledge, we describe the only reported case of acute posterior tibial nerve compression resulting from adjacent haemotogenous pyogenic calcaneal osteomyelitis.

**Case presentation:**

A previously healthy 38-year-old Caucasian woman developed symptoms of acute tarsal tunnel syndrome in her right foot over a six-day period. No antecedent trauma or systemic symptoms were noted. Magnetic resonance imaging and bone scan imaging, followed by surgical decompression and bone biopsy confirmed a diagnosis of *Staphylococcus aureus *calcaneal osteomyelitis. Her pain and paraesthesia disappeared after the operation, while her inflammatory markers normalised during a 12-week course of antibiotics. After four years she has remained asymptomatic without any indication of recurrence.

**Conclusion:**

This case is not just unique in describing osteomyelitis as a cause of tarsal tunnel syndrome, because haemotogenous calcaneal osteomyelitis is in itself a rare pathology. We recommend considering infection as a differential diagnosis in patients presenting with acute tarsal tunnel syndrome.

## Introduction

The tarsal tunnel is a fibro-osseous space bounded by the flexor retinaculum, posterior talus and the calcaneus, as well as the anterior medial malleolar artery. Various anatomical compartmental divisions have been described [1]. The tarsal tunnel houses the tendons of tibialis posterior, flexor digitorum longus and flexor hallucis longus, as well as the posterior tibial nerve, artery and vein.

Tarsal tunnel syndrome is an entrapment neuropathy of one or all branches of the posterior tibial nerve (lateral plantar, medial plantar and medial calcaneal nerves) and was first described independently by Keck and Lam in 1962 [2,3]. Various aetiologies for tarsal tunnel syndrome have been previously described, but here we describe its first known case of unique association with osteomyelitis.

## Case presentation

We present the case of a previously healthy 38-year-old Caucasian woman who presented to our hospital's emergency department with a six-day history of severe sharp and burning right heel and foot pain with inability to bear weight. She had no antecedent trauma or systemic symptoms. The pain was referred distally along the medial and lateral plantar aspect of her foot into the toes, with exacerbation at night and with ambulation.

A physical examination revealed a warm localised swelling around her medial malleolus with no overlying erythema. Her ankle movements were normal but her subtalar joint movement was painful and restricted. A neurological examination confirmed altered sensation over the plantar surface of her foot and toes. Tinel's sign was also noted to be absent along the course of her posterior tibial nerve.

**Figure 1:**
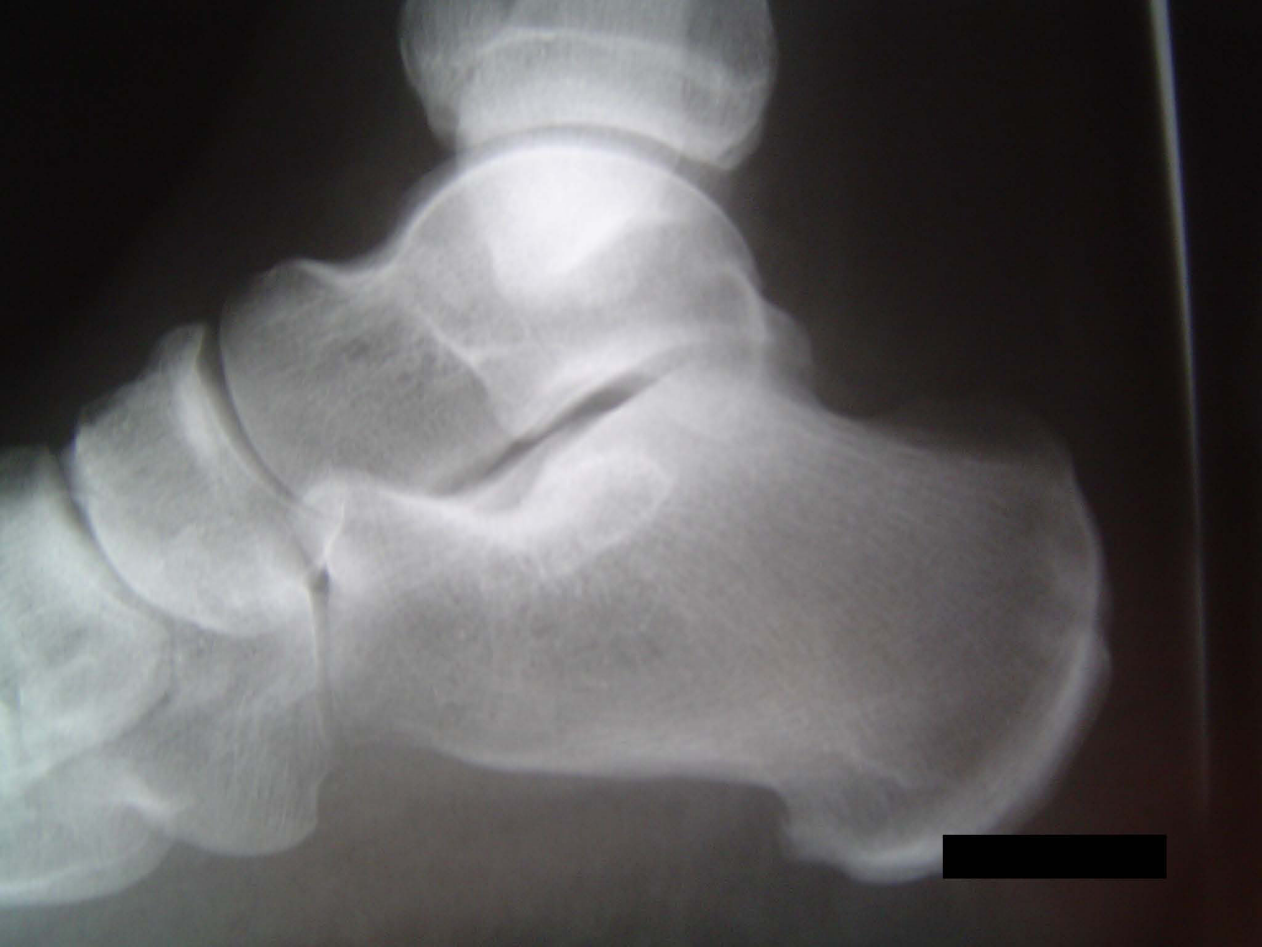
**Normal plain radiograph at presentation**.

**Figure 2:**
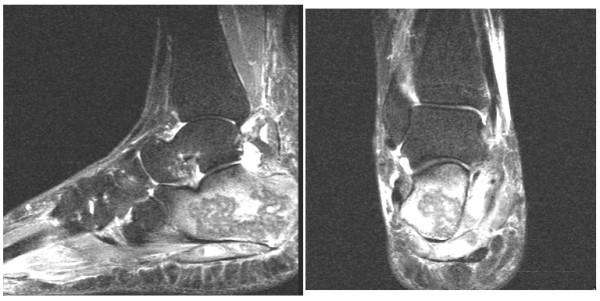
**Saggital and coronal T2/STIR-weighted magnetic resonance scan of the foot demonstrating calcaneal edema and edema within the tarsal tunnel, with a tense adjacent subtalar joint effusion**.

**Figure 3:**
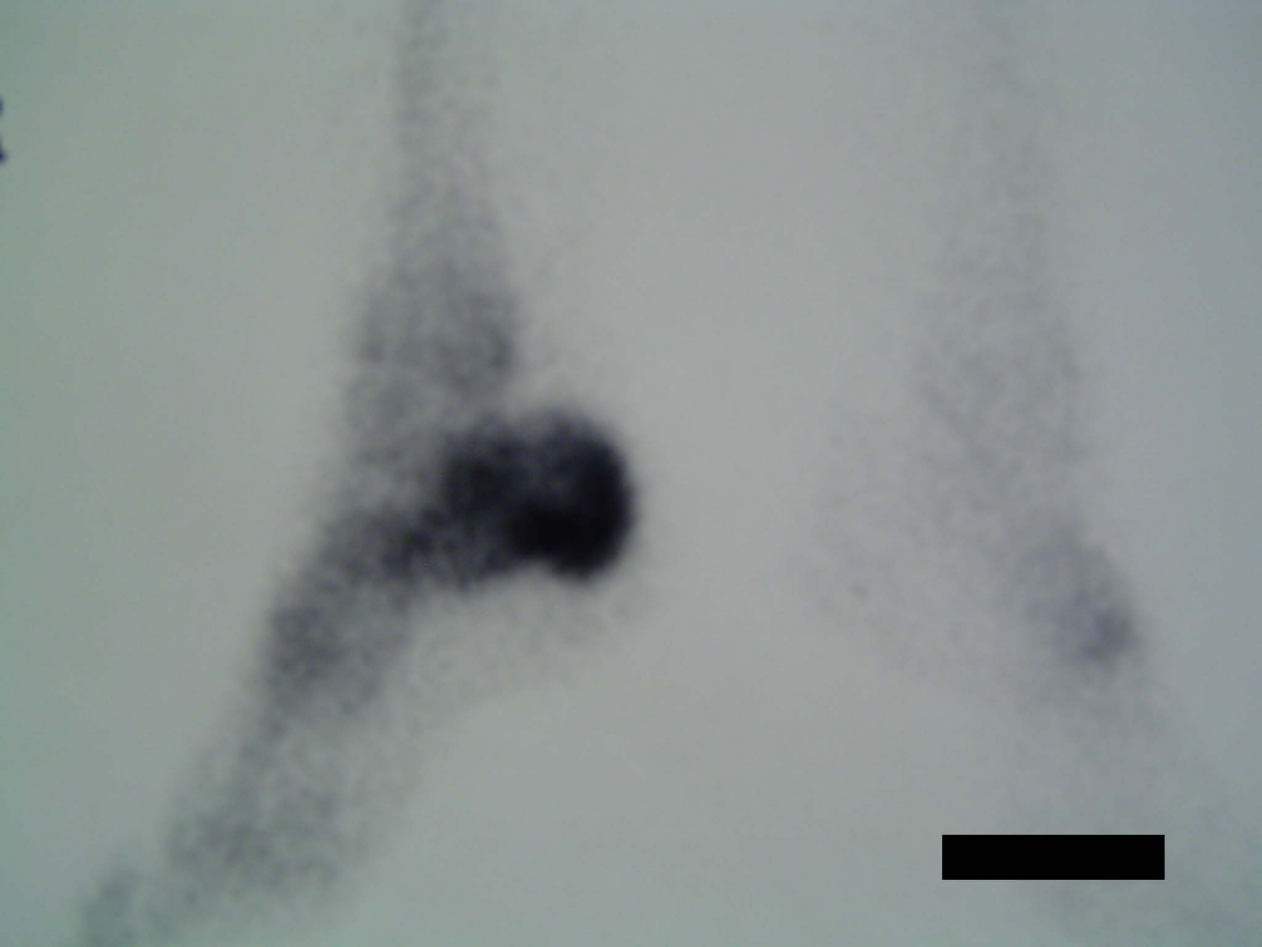
**Bone scan showing increased calcaneal tracer uptake**.

Our patient's inflammatory markers were raised (white cell count at 12.2 × 10^9^cells/L [neutrophils = 11.0] C-reactive protein at 194 and erythrocyte sedimentation rate at 59), while her autoimmune antibody titres and blood cultures were found to be normal. Initial plain radiographs were unremarkable (Figure 1), while a magnetic resonance imaging (MRI) of her hindfoot demonstrated an increased calcaneal signal intensity on the T2/STIR-weighted images, with a tense effusion of the subtalar joint (Figure 2). Bone scanning confirmed the presence of isolated increased uptake of radioisotope in the calcaneus on blood pool and delayed phases (Figure 3).

In view of the radiological and clinical evidence available, early exploration of the tarsal tunnel was performed via a posteromedial approach. The operative findings were of widespread oedema adjacent to the medial wall of the calcaneus extending into the proximal end of the tarsal tunnel. The posterior tibial nerve itself was noted to be erythematous and swollen. Following decompression of the tarsal canal, a core needle bone biopsy of the body of the calcaneus was performed. Postoperatively, our patient's pain improved and her neurological symptoms resolved within 24 hours. Intraoperative bone biopsy microbiology grew *Staphylococcus aureus *sensitive to vancomycin. She was subsequently treated for 12 weeks with a combination of oral and intravenous antibiotics and made a full recovery with normal inflammatory markers at six months postoperatively. When she was followed up after four years, she was noted to have remained asymptomatic with no indication of recurrence of the disease.

## Discussion

Tarsal tunnel syndrome is an uncommon condition [4]. Its aetiologies have been classified as idiopathic, extrinsic, intrinsic or tension-related. Extrinsic causes include local trauma (fractures, dislocations, sprains or crush), or local bony prominence pressure. Intrinsic causes comprise space-occupying lesions (ganglia, tumour or hematoma), intrinsic neuropathy, and venous plexus congestion [5-7]. Tension secondary to hindfoot valgus has also been demonstrated as an isolated source, as well as exacerbating other causes [8]. Unusual causes that have been previously described include secondary to a partial rupture of the flexor hallucis longus tendon [9], amyloidoma following haemodialysis [10], and angioleiomyoma [11]. An underlying pathology is absent in up to 50% of cases [12].

To the best of our knowledge, the case we report is the only recorded occurrence of tarsal tunnel syndrome resulting from an adjacent pyogenic calcaneal osteomyelitis. Our patient responded well to debridement and decompression of her tarsal tunnel.

## Conclusion

This case is not just unique in describing osteomyelitis as a rare cause of tarsal tunnel syndrome, but also in describing the occurrence of isolated acute haemotogenous osteomyelitis of the calcaneus, which in itself is rarely described in adults [13]. We recommend considering infection along with all the other described aetiologies for the differential diagnosis of acute tarsal tunnel syndrome.

## Consent

Written informed consent was obtained from the patient for publication of this case report and any accompanying images. A copy of the written consent is available for review by the Editor-in-Chief of this journal.

## Competing interests

The authors declare that they have no competing interests.

## Authors' contributions

DB collated the clinical data and wrote the manuscript. SS collected the radiological data and assisted in proofreading the manuscript. RD was the senior surgeon who undertook the surgical management and care of the patient. All authors read and approved the final manuscript.
